# A Criterion for the Complete Deposition of Magnetic Beads on the Walls of Microchannels

**DOI:** 10.1371/journal.pone.0151053

**Published:** 2016-03-23

**Authors:** Jordi Pallares

**Affiliations:** Department of Mechanical Engineering, Universitat Rovira i Virgili, Tarragona, Spain; virginia commonwealth university, UNITED STATES

## Abstract

This paper analyzes numerical simulations of the trajectories of magnetic beads in a microchannel, with a nearby permanent cubical magnet, under different flow and magnetic conditions. Analytically derived local fluid velocities and local magnetic forces have been used to track the particles. A centered position and a lateral position of the magnet above the microchannel are considered. The computed fractions of deposited particles on the walls are compared successfully with a new theoretically derived criterion that imposes a relation between the sizes of the magnet and the microchannel and the particle Stokes and Alfvén numbers to obtain the complete deposition of the flowing particles on the wall. In the cases in which all the particles, initially distributed uniformly across the section of the microchannel, are deposited on the walls, the simulations predict the accumulation of the major part of particles on the wall closest to the magnet and near the first half of the streamwise length of the magnet.

## Introduction

The application of magnetism in fluidic microsystems has been used since the boom of microfluidics in the early 2000 [1] with the concept of micro total analysis systems. Magnetic forces can be used to manipulate magnetic objects as magnetic particles or magnetically labelled cells. The movement of magnetic beads in microfluidic applications is usually controlled by permanent magnets or by magnetic fields generated by electric currents. Magnetohydrodynamic pumps, based on the generation of a magnetic field perpendicular to an electric field, can be used, as an alternative to pressure driven or electroosmotic flows, to produce flow in a conducting fluid [2, 3]. The same principle can be used for mixing [4, 5]. Plugs of ferrofluid can be moved with magnets to induce or to block the flow of an immiscible liquid [6, 7]. More information about these few examples and other applications involving magnetic forces in microflows can be found, for example, in the reviews of Gijs et al. [8], Pamme [9], Berthier and Silberzan [10] and, recently, in Yang et al [11].

Microsized magnetic beads are constituted by nanoparticles of iron oxides embedded in a biologically-compatible polymer (latex or polystyrene) sphere. The external surface of the sphere can be coated with biological molecules such as enzymes or DNA fragments which can be easily transported, by applying a magnetic field, towards specific locations or biological targets. Magnetic beads are used mostly for in vitro applications (biodiagnostics and biorecognition) and recently for in vivo applications, such as cancer treatment. In this case functionalized magnetic particles can by transported by the blood flow and retained by a magnet implemented in the treatment zone. For the most common applications the sizes of the magnetic particles range from 5 nm to 6 μm. The smallest particles (5 nm- 100 nm) can be used for applications in which the particles interact with proteins, viruses and genes [12]. Magnetic beads used for biotechnology are usually superparamagnetic because it is desired that the magnetic force vanish when the externally imposed magnetic field is switched off.

This study is focused on the prediction of the trajectories of magnetic beads in microchannels and the identification of the required magnetic and relevant physical properties needed for the complete deposition of the magnetic beads on the walls of the channel.

## Physical and Mathematical Model

**Fig 1:**
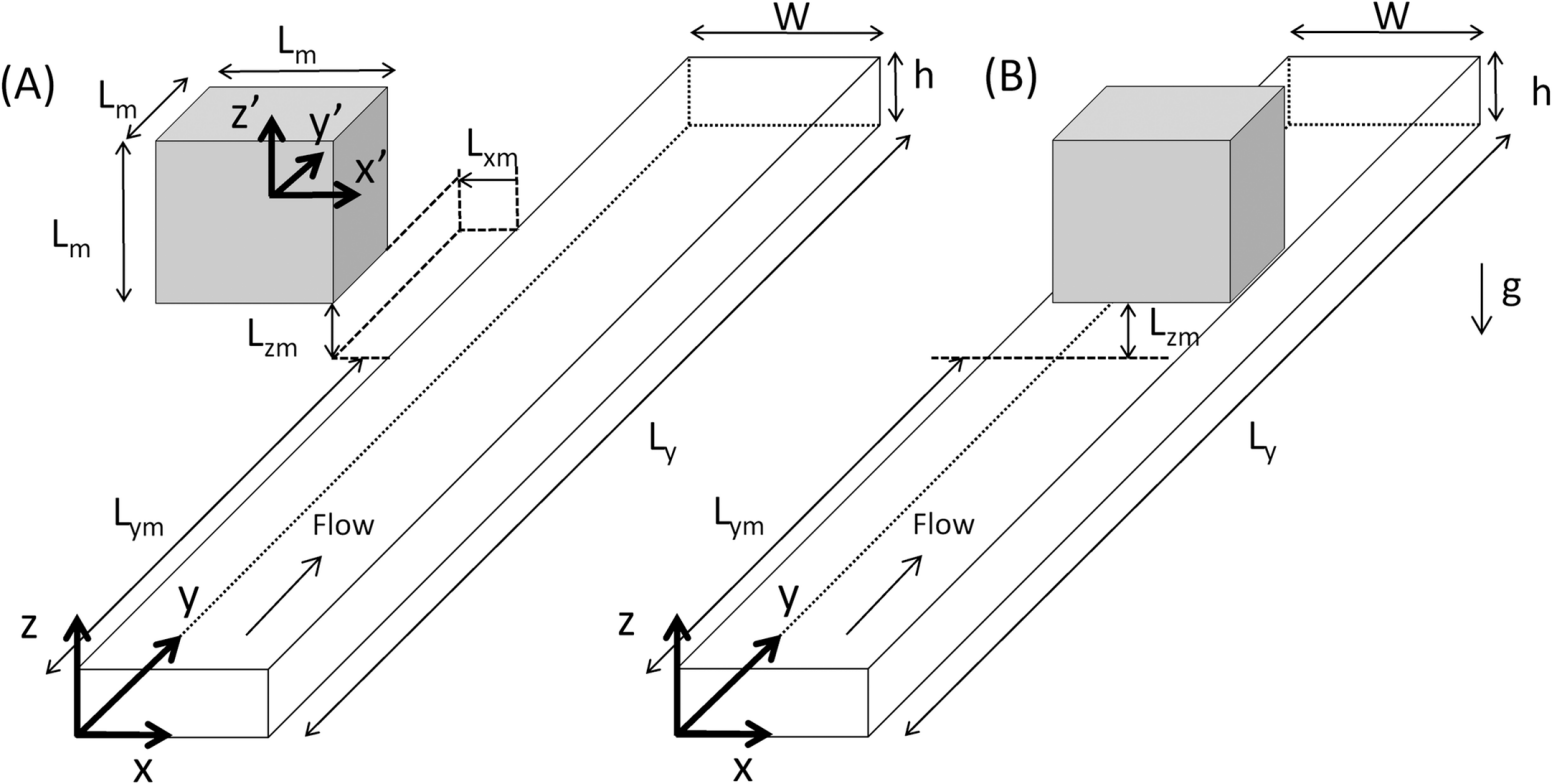
Physical model and coordinate systems.

The physical model is shown in Fig 1. A permanent cubical magnet is located above a straight microchannel with rectangular cross section. The distance between the top wall of the microchannel and the bottom of the magnet is *L*_*zm*_. Two different positions of the magnet have been considered. A lateral position *L*_*xm*_ = 0 (see Fig 1A) and a centered position for which the magnet is located symmetrically at the center of the microchannel (see Fig 1B). Note that the lateral position generates a weaker magnetic field within the channel than the centered position but, in experiments, it allows the visual inspection of the region close to the magnet where magnetic beads are expected to deposit. In both cases the gravity vector acts along the negative *z* direction.

The flow is assumed to be incompressible, laminar, steady and the physical properties of the fluid constant. The particles are assumed to be spherical and they do not affect the flow according to the dilute approximation (i.e. one-way coupled Eulerian-Lagrangian approach). Under these hypotheses the flow can be described by the closed solution of the axial momentum equation for the pressure-driven fully developed flow. The axial velocity distribution, scaled with the averaged velocity, within the cross section of the microchannel is given by Eq 1 (see for example Shah and London [13])

$$v^{*}=\frac{48}{\pi^{3}}\frac{\sum_{n=1,3,\ldots }^{\infty }\frac{{\left(-1\right)}^{\left(n-1\right)/2}}{n^{3}}\left[1-\frac{\cos h\left(n\pi \left(z-\frac{h}{2}\right)/W\right)}{\cos h\left(n\pi h/2W\right)}\right]cos\left(n\pi \left(x-\frac{W}{2}\right)/W\right)}{\left[1-\frac{192}{\pi^{5}}\left(\frac{W}{h}\right)\sum_{n=1,3,\ldots }^{\infty }\frac{1}{n^{5}}\tan h\left(n\pi h/2W\right)\right]}$$ (1)

The particles are tracked with a Lagrangian approach based on the numerical solution of the kinematic equation and the force balance equation for each particle. The non-dimensional forms of these equations can be written as,

$$\frac{d{\vec{r}}_{p}^{*}}{dt^{*}}={\vec{V}}_{p}^{*}$$ (2)

$$\frac{d{\vec{V}}_{p}^{*}}{dt^{*}}=\frac{1}{Fr^{2}}\frac{\vec{g}}{g}-\frac{3}{4}C_{D}\frac{\rho_{f}}{\rho_{p}}\frac{1}{d_{p}^{*}}\left|{\vec{V}}_{p}^{*}-{\vec{V}}^{*}\right|\left[{\vec{V}}_{p}^{*}-{\vec{V}}^{*}\right]+\frac{\chi \mu_{o}M_{s}^{2}}{{\left(4\pi \right)}^{2}\rho_{p}{\bar{V}}^{2}}\left({\vec{B}}^{*}\cdot {\vec{\nabla }}^{*}\right){\vec{B}}^{*}$$ (3)

The length and velocity scales used to obtain the non-dimensional variables are the height of the microchannel (*h*) and the average velocity of the flow ($\bar{V}$). The three terms on the right hand side of Eq 3 are the gravity force, the drag force and the magnetic force. For micron-sized particles suspended in liquid laminar flows the Stokes number (*St*) and the particle Reynolds number (*Re*_*p*_) are much lower than unity and the drag coefficient can be computed as *C*_*D*_ = 24/*Re*_*p*_. Simulations carried out with the addition of the lift force to Eq 3 at the largest Reynolds number considered (*Re* = 10) indicate that this force [14] can be neglected because trajectories are not affected significantly by the inclusion of this term. As a first approximation, the near-wall drag modification [15] or the lubrication force [16] has been neglected.

The form of the magnetic term in Eq 3 is obtained from the dimensional form of the force per unit mass given in Eq 4 [17], in which the initial magnetization of the particles is neglected [18].

$$\frac{{\vec{F}}_{m}}{m_{p}}=\frac{\chi }{\mu_{o}\rho_{p}}\left(\vec{B}\cdot \vec{\nabla }\right)\vec{B}$$ (4)

Eq 4 assumes a linear dependence of magnetization of the particle with the applied magnetic field (see for example [18]). This approximation is valid for magnetic fields small enough to avoid the magnetic saturation of the particles. The magnetization of superparamagnetic particles, or their magnetization curve, can be modeled with the Langevin’s law [10] that relates the degree of saturation as a function of the applied magnetic field. Typical magnetization curves show saturation of the particles between 3 A m^2^/kg [18] and 68 A m^2^/kg [19] with large variability depending on the size and specific composition of the particle.

The magnetic flux density has been computed using the analytical solution for permanent rectangular magnets reported by Furlani [20]. The components of the magnetic flux density are indicated in Eqs 5 to 7.

$$B_{x}^{*}=B_{x}\frac{4\pi }{\mu_{o}M_{s}}=\sum_{k=1}^{2}\sum_{n=1}^{2}{\left(-1\right)}^{k+n}\;\log\left[F\left(x\prime ,y\prime ,z\prime ,x_{n}^{\prime },y_{1}^{\prime },y_{2}^{\prime },z_{k}^{\prime }\right)\right]$$ (5)

$$B_{y}^{*}=B_{y}\frac{4\pi }{\mu_{o}M_{s}}=\sum_{k=1}^{2}\sum_{n=1}^{2}{\left(-1\right)}^{k+n}\;\log\left[H\left(x\prime ,y\prime ,z\prime ,x\prime_{1},x\prime_{2},y\prime_{n},z\prime_{k}\right)\right]$$ (6)

$$B_{z}^{*}=B_{z}\frac{4\pi }{\mu_{o}M_{s}}=\sum_{k=1}^{2}\sum_{n=1}^{2}\sum_{l=1}^{2}{\left(-1\right)}^{k+n+l}\;\tan^{-1}\left[G\left(x\prime ,y\prime ,z\prime ,x_{n}^{\prime },y\prime_{l},z_{k}^{\prime }\right)\right]$$ (7)

where *F*, *H* and *G* are defined as,

$$F\left(x^{\prime },y^{\prime },z^{\prime },x_{n}^{\prime },y_{1}^{\prime },y_{2}^{\prime },z_{k}^{\prime }\right)=\frac{\left(y^{\prime }-y_{1}^{\prime }\right){\left[{\left(x^{\prime }-x_{n}^{\prime }\right)}^{2}+{\left(y^{\prime }-y_{1}^{\prime }\right)}^{2}+{\left(z^{\prime }-z_{k}^{\prime }\right)}^{2}\right]}^{1/2}}{\left(y^{\prime }-y_{2}^{\prime }\right){\left[{\left(x^{\prime }-x_{n}^{\prime }\right)}^{2}+{\left(y^{\prime }-y_{2}^{\prime }\right)}^{2}+{\left(z^{\prime }-z_{k}^{\prime }\right)}^{2}\right]}^{1/2}}$$ (8)

$$H\left(x^{\prime },y^{\prime },z^{\prime },x_{1}^{\prime },x_{2}^{\prime },y_{n}^{\prime },z_{k}^{\prime }\right)=\frac{\left(x^{\prime }-x_{1}^{\prime }\right){\left[{\left(x^{\prime }-x_{1}^{\prime }\right)}^{2}+{\left(y^{\prime }-y_{n}^{\prime }\right)}^{2}+{\left(z^{\prime }-z_{k}^{\prime }\right)}^{2}\right]}^{1/2}}{\left(x^{\prime }-x_{2}^{\prime }\right){\left[{\left(x^{\prime }-x_{2}^{\prime }\right)}^{2}+{\left(y^{\prime }-y_{n}^{\prime }\right)}^{2}+{\left(z^{\prime }-z_{k}^{\prime }\right)}^{2}\right]}^{1/2}}$$ (9)

$$G\left(x^{\prime },y^{\prime },z^{\prime },x_{n}^{\prime },y_{l}^{\prime },z_{k}^{\prime }\right)=\frac{\left(x^{\prime }-x_{n}^{\prime }\right)\;\left(y^{\prime }-y_{l}^{\prime }\right)}{\left(z^{\prime }-z_{k}^{\prime }\right)\;{\left[{\left(x^{\prime }-x_{n}^{\prime }\right)}^{2}+{\left(y^{\prime }-y_{l}^{\prime }\right)}^{2}+{\left(z^{\prime }-z_{k}^{\prime }\right)}^{2}\right]}^{1/2}}$$ (10)

The system of reference, indicated with the prime symbol, used for Eqs 5 to 10 has its origin in the center of mass of the magnet (see Fig 1) and $\left(x_{1}^{\prime },y_{1}^{\prime },z_{1}^{\prime }\right)$ and $\left(x_{2}^{\prime },y_{2}^{\prime },z_{2}^{\prime }\right)$ are the coordinates of two diagonally opposed vertices of the magnet which has the magnetization aligned with the z’ direction. For example, for a cubical magnet of size *L*_*m*_, $x_{1}^{\prime }=y_{1}^{\prime }=z_{1}^{\prime }=-L_{m}/2$ and $x_{2}^{\prime }=y_{2}^{\prime }=z_{2}^{\prime }=L_{m}/2$.

Eqs 2 and 3 were numerically integrated using the second order Crank-Nicolson scheme. The Lagrangian tracking in-house code used here, developed in FORTRAN language and parallelized using OpenMP directives, has been previously applied for the simulation of the turbulent dispersion of particles in forced [21] and natural convection [22] flows.

The fluid velocity at the position of the particle was computed using Eq 1. In Eq 3, the term $\left({\vec{B}}^{*}\cdot {\vec{\nabla }}^{*}\right){\vec{B}}^{*}$, at the particle position, has been computed analytically. The exact expressions for the three components were derived using *Mathematica* [23] and the output obtained with the FortranForm command was directly copied and pasted in the simulation code to avoid errors. A typical time step for the simulations was Δt* = 10^−3^.

The computational domain for the lateral position of the magnet had dimensions $L_{x}^{*}=5,\;L_{y}^{*}=100,\;L_{z}^{*}=1$, while half of the microchannel was considered for the centered position because of symmetry with respect to the plane x* = 2.5 (see Fig 1B). In this case the dimensions were $L_{x}^{*}=2.5,\;L_{y}^{*}=100,\;L_{z}^{*}=1$. The computational domain was divided into 49x499x19 equal volumes for the lateral case and into 99x499x19 equal volumes for the centered case. A particle was placed at the center of each volume located at the inlet of the channel (y* = 0) (i.e. 49x19 = 931 particles for the lateral case and 99x19 = 1881 particles for the centered case). The time marching scheme was initialized setting the velocity of the particle equal to that of the fluid at the specific location of the particle. The positions of the particles were stored and the instantaneous concentration of particles was determined by computing the number of particles in each volume.

This information was used to calculate the joint conditional probability for a particle to be at location *x*, *y*, *z* at time *t*, given that the particle was released at the position *x*_0_, *y*_0_ = 0, *z*_0_ at time *t*_0_ = 0. This probability can be used to extract information about the behavior of a continuous sources of particles located at the inlet of the channel. A similar approach is used in the simulation of scalar dispersion in turbulent flows at high Schmidt numbers [24]. This procedure based, on the Lagrangian tracking, overcomes the use of very fine grids, needed by the Eulerian approach, to capture the thin mixing interface of scalars (or clouds of particles) with very low molecular (or Brownian) diffusivity. For example, for 1 micron particles the Brownian diffusivity [10] at ambient temperature is 4·10^−13^ m^2^/s and the corresponding Schmidt number is 2·10^6^. Even for the 5 nm particles the Schmidt number is about 10^4^, which makes the numerical solution of the transport equation for the concentration of particles computationally very expensive using the conventional Eulerian approach.

## Results and Discussion

As suggested by Eq (3), the trajectories of the particles are dominated by the drag force, that is proportional to ${\left(d_{p}^{*}\right)}^{-2}$ and by the magnetic force, proportional to the non-dimensional group *M*, defined in Eq 11 and to the non-dimensional term $\left({\vec{B}}^{*}\cdot {\vec{\nabla }}^{*}\right){\vec{B}}^{*}$.

$$M=\frac{\chi \mu_{o}M_{s}^{2}}{{\left(4\pi \right)}^{2}\;\rho_{p}{\bar{V}}^{2}}$$ (11)

The first contribution to the magnetic force is the group *M* that depends on the magnetic characteristic of the particles (*χ*) and on the magnetization of the magnet. The second contribution, the term $\left({\vec{B}}^{*}\cdot {\vec{\nabla }}^{*}\right){\vec{B}}^{*}$, depends only on the size of the magnet and its relative position with respect to the microchannel.

**Fig 2:**
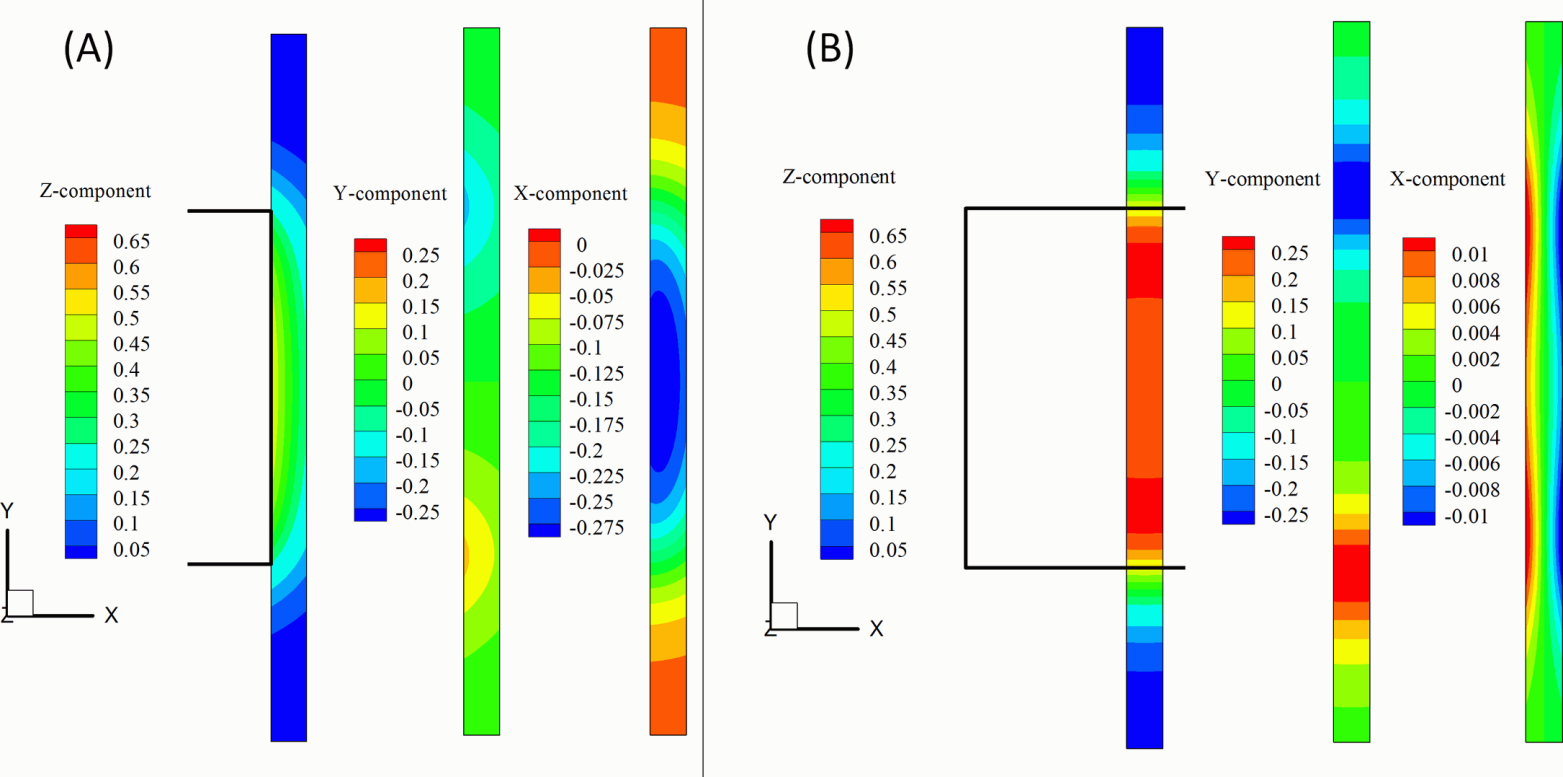
Contours of the three components of the term $\left({\vec{B}}^{*}\cdot {\vec{\nabla }}^{*}\right){\vec{B}}^{*}$. (A) Lateral position (see Fig 1A). (B) Central position. The relative position of the magnet (L_m_ = 5 mm) with respect to the microchannel (h = 0.1 mm W = 0.5 mm) is indicated by the black lines.

As an example of the spatial distribution of the force generated by a magnet, Fig 2 shows the contours of the three components of the term $\left({\vec{B}}^{*}\cdot {\vec{\nabla }}^{*}\right){\vec{B}}^{*}$ for the lateral (Fig 2A) and centered (Fig 2B) position of the magnet. The field corresponds to a cubical magnet (*L*_*m*_ = 5 *mm*) located at *L*_*zm*_ = 1 *mm*, above a microchannel (*h* = 0.1 *mm*, *W* = 0.5 *mm*). This distance is the typical width of the plastic cover of the microchips. The plots correspond to top views (*x-y* plane) of the microchannel. It can be seen that the lateral configuration (Fig 2A) generates significant vertical (*z*) and lateral (*x*) forces towards the magnet. The centered case (Fig 2B) generates intense vertical forces with maxima located close to the edges of the magnet and maximum axial (*y*) forces near the edges of the magnet.

To estimate the size and characteristics of the magnet needed to capture the magnetic beads which are flowing within a fluid we assume that the magnet is located at the top of the microchannel with a size larger or equal to the width of the microchannel.

The vertical velocity induced by the magnet can be estimated from the steady state vertical force balance on the particle. (see Eq 3)

$$\frac{dw^{*}}{dt^{*}}=-Fr^{-2}-\frac{w^{*}}{St}+M\left({\vec{B}}^{*}\cdot {\vec{\nabla }}^{*}\right)B_{z}^{*}=0$$ (12)

Rearranging Eq 12, the dimensional vertical velocity can be written as,

$$w=\bar{V}\;St\left(-Fr^{-2}+M\left({\vec{B}}^{*}\cdot {\vec{\nabla }}^{*}\right)B_{z}^{*}\right)$$ (13)

The particles near the bottom wall of the channel need to travel vertically a distance *h* to reach the top wall of the channel, which is located below the magnet. If the vertical velocity is given by Eq 13, then the time for the vertical travel is

$$t_{z}=\frac{h}{\bar{V}}\;\frac{1}{St\left(-Fr^{-2}+M\left({\vec{B}}^{*}\cdot {\vec{\nabla }}^{*}\right)B_{z}^{*}\right)}$$ (14)

To verify that all the particles are captured by the magnet we can consider that the particles located at the bottom wall of the channel should not travel along the streamwise direction a distance larger than the length of the magnet during the period of time given by Eq 14. If we take the average flow velocity, as an upper limit, an estimation of the length of the magnet ($L_{m}=t_{z}\bar{V}$) can be written as,

$$L_{m}\approx \frac{h}{St\left(-Fr^{-2}+M\left({\vec{B}}^{*}\cdot {\vec{\nabla }}^{*}\right)B_{z}^{*}\right)}$$ (15)

**Fig 3:**
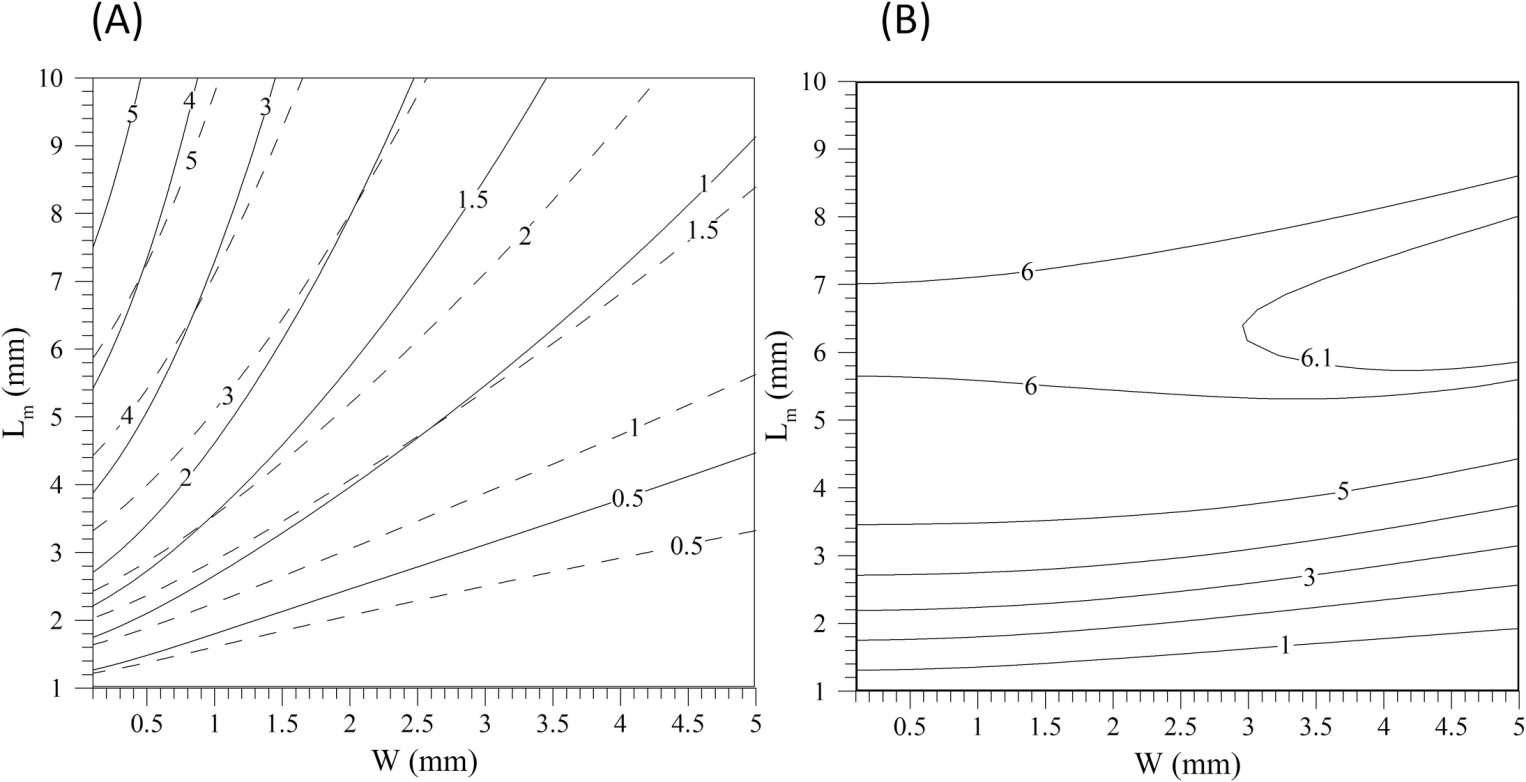
Volume averaged modulus of some components of the vector $\left(\left({\vec{B}}^{*}\cdot {\vec{\nabla }}^{*}\right){\vec{B}}^{*}\right)$. (A) the lateral position (see Fig 1A) (B) the centered position (see Fig 1B). The continuous lines correspond to the volume averaged modulus of the vertical component (i.e. $\left|\left({\vec{B}}^{*}\cdot {\vec{\nabla }}^{*}\right)B_{z}^{*}\right|$) and the dashed line contours to the modulus of the vector defined by the sum of the vertical and lateral components, ${\left[{\left(\left({\vec{B}}^{*}\cdot {\vec{\nabla }}^{*}\right)B_{z}^{*}\right)}^{2}+{\left(\left({\vec{B}}^{*}\cdot {\vec{\nabla }}^{*}\right)B_{x}^{*}\right)}^{2}\right]}^{1/2}$.

The magnitude of the term $\left({\vec{B}}^{*}\cdot {\vec{\nabla }}^{*}\right)B_{z}^{*}$ needs to be evaluated for the particular shape, size and location of the magnet. Fig 3 shows the magnitude of this term for different sizes of cubical magnets (*L*_*m*_) located at the top of the microchannel of width W. The height of the microchannel is *h* = 100 μm and the bottom of the magnet is 1mm above the top wall of the microchannel. The value has been averaged in the volume of the microchannel below the magnet. It can be seen that the average value of this term for the lateral configuration depends strongly on the aspect ratio of the microchannel while for the centered case this dependence is smaller. In the case of the lateral position of the magnet and for comparison purposes, we include in Fig 3A the volume averaged values of the modulus of the sum of the vertical and lateral component of the vector $\left(\left({\vec{B}}^{*}\cdot {\vec{\nabla }}^{*}\right){\vec{B}}^{*}\right)$, (i.e. ${\left[{\left(\left({\vec{B}}^{*}\cdot {\vec{\nabla }}^{*}\right)B_{z}^{*}\right)}^{2}+{\left(\left({\vec{B}}^{*}\cdot {\vec{\nabla }}^{*}\right)B_{x}^{*}\right)}^{2}\right]}^{1/2}$) because both components contribute to drive the particle towards the magnet. It can be seen that the use in of the volume averaged value of $\left|\left({\vec{B}}^{*}\cdot {\vec{\nabla }}^{*}\right)B_{z}^{*}\right|$, instead of ${\left[{\left(\left({\vec{B}}^{*}\cdot {\vec{\nabla }}^{*}\right)B_{z}^{*}\right)}^{2}+{\left(\left({\vec{B}}^{*}\cdot {\vec{\nabla }}^{*}\right)B_{x}^{*}\right)}^{2}\right]}^{1/2}$ in Eq 14 gives a more conservative estimation of the size of the magnet.

For cubical magnets with sizes between 4 mm and 10 mm the term $\left|\left(\vec{B}\cdot \vec{\nabla }\right)B_{z}\right|$ is in the range 5000 m^-1^–6000 m^-1^. Additionally if we consider that the typical values of the magnetic saturation *M*_*s*_ for permanent magnets are between 5·10^5^–10^6^ A/m (*B*_*s*_ = 0.6–1.5 T) and that 0.1 ≤ *χ* ≤ 1 [18], *ρ*_*p*_ ≈ 1600 kg/m^3^ and *h*≈100 μm the contribution of the gravity force can be neglected in comparison with the magnetic force and Eq 15 can be written as

$$\frac{L_{m}}{h}\approx \frac{1}{St\;\left(M\left({\vec{B}}^{*}\cdot {\vec{\nabla }}^{*}\right)B_{z}^{*}\right)}$$ (16)

Eq 16 indicates that the non-dimensional size of the magnet needed to capture the particles depends on the particle Stokes number and on the term $M\left({\vec{B}}^{*}\cdot {\vec{\nabla }}^{*}\right)B_{z}^{*}$ that represents the ratio between the applied local magnetic force and the particle inertia ($\rho_{p}{\bar{V}}^{2}$). This ratio is known in magnetohydrodynamics as the Alfvén number, $Al=B^{2}/\mu_{o}\rho {\bar{V}}^{2}$ (see for example Lee and Choi [25]). Similarly we can introduce and define the particle Alfvén number as,

$$Al_{p}=\frac{\frac{\chi \mu_{o}M_{s}^{2}}{{\left(4\pi \right)}^{2}}\langle \left({\vec{B}}^{*}\cdot {\vec{\nabla }}^{*}\right)B_{z}^{*}\rangle }{\rho_{p}{\bar{V}}^{2}}=M\langle \left({\vec{B}}^{*}\cdot {\vec{\nabla }}^{*}\right)B_{z}^{*}\rangle$$ (17)

using the volume-averaged magnetic force in the microchannel below the magnet (i.e. $M\langle \left({\vec{B}}^{*}\cdot {\vec{\nabla }}^{*}\right)B_{z}^{*}\rangle$. This quantity can be computed with Eqs 5 to 10 or obtained from Fig 3 for the specific conditions considered in this figure. Eq 16 can be rewritten as,

$$\frac{L_{m}}{h}\approx \frac{1}{St\;Al_{p}}$$ (18)

Under physical conditions expressed above and at $Re=\bar{V}h/\nu =1,\;(\bar{V}\approx 1\;mm/s)$, the lengths predicted by Eq 16 for *M*_*s*_ = 5·10^5^ A/m and 10^6^ A/m are 1.5 mm and 0.4 mm, respectively for *χ* = 1, and 15 mm and 3.8 mm, for *χ* = 0.1. This variability of the sizes, depending on the magnetic properties of the magnet (*M*_*s*_) and of the particles (*χ*), suggests that the selection of the size of the magnet should be carried out with the knowledge of these properties.

In these particular examples the maximum degree of saturation of the particles depends on the maximum value of the magnetic field within the channel, which is located at the top wall. Specifically for *M*_*s*_ = 5·10^5^ A/m (*L*_*m*_ = 1.5 mm) the maximum values of the magnetic field for the centered position of the magnet and for the lateral position are 0.06 T and 0.04 T, respectively. The magnetization of the particle can be considered linear in the range of magnetic field from 0 to these maximum values depending on the particular magnetization curve of the particles, which some manufacturers of particles facilitate. For the present conditions, the linear dependence of the magnetization on the magnetic field for 0<*B* < 0.06 T is a reasonable approximation (as shown in Fig 7 of [19], Fig 5 of [26], Fig 4 of [27]) that correspond to particles of different materials and sizes.

**Fig 4:**
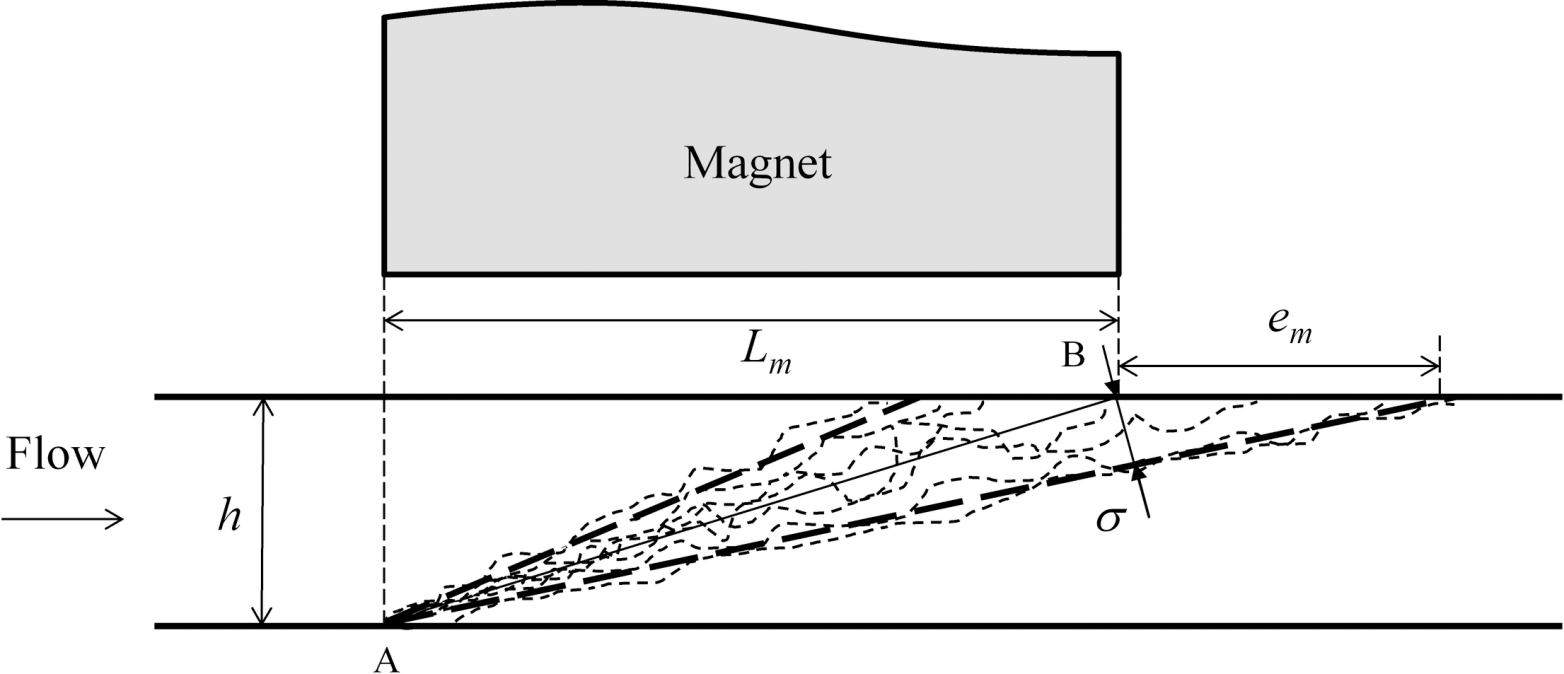
Sketch of the paths of a particle located at the bottom of the microchannel (point A).

The criterion expressed in Eq 18 is valid for magnetic particles with negligible Brownian motion. It is known that trajectories of nanoparticles can be affected by thermal random fluctuations [28–30]. Fig 4 illustrates a sketch of the possible paths of a particle initially located at the bottom of the channel (point A). The effect of the Brownian motion, which is indicated by the standard deviation, *σ*, of the displacements around a mean path (see Fig 4), can be handled by an extension of the size of the magnet of *e*_*m*_ to capture the fraction of particles that would reach the top wall downwind the trailing edge of the magnet. According to the sketch shown in Fig 4, the standard deviation of the paths can be related with the two-dimensional geometry of the problem as,

$$\sigma =\frac{h\;e_{m}}{\sqrt{h^{2}+{\left(L_{m}+e_{m}\right)}^{2}}}$$ (19)

The standard deviation of the random displacements can be associated with the Brownian diffusion coefficient (*D*_*Br*_ = *k*_*B*_ *T*/3*πμd*_*p*_) and the time for the vertical travel of the particle as,

$$\sigma \approx \sqrt{2\;D_{Br}\;t_{z}}$$ (20)

The substitution of Eq 14 into Eq 20 and using Eq 19 leads to,

$$\frac{e_{m}^{2}}{h^{2}+{\left(L_{m}+e_{m}\right)}^{2}}\approx \frac{192\pi \;k_{B}\;T}{d_{p}^{3}\;\chi \mu_{o}M_{s}^{2}\langle \left({\vec{B}}^{*}\cdot {\vec{\nabla }}^{*}\right)B_{z}^{*}\rangle }$$ (21)

where the effect of gravity has been neglected (i.e. *Fr*^−2^ = 0). Eq 21 relates the extension of the magnet required, given the characteristics of the particles and the dimensions of the channel, to capture the particles that have been drifted along the streamwise direction by the action of the Brownian motion. If the dimension of the magnet is much larger than the extension (*L*_*m*_ ≫ *e*_*m*_) and the channel height (*L*_*m*_ ≫ *h*), Eq 21 can be rewritten as,

$$\frac{e_{m}}{L_{m}}\approx {\left(\frac{192\pi \;k_{B}\;T}{d_{p}^{3}\;\chi \mu_{o}M_{s}^{2}\langle \left({\vec{B}}^{*}\cdot {\vec{\nabla }}^{*}\right)B_{z}^{*}\rangle }\right)}^{1/2}$$ (22)

As an example, considering $\langle \left({\vec{B}}^{*}\cdot {\vec{\nabla }}^{*}\right)B_{z}^{*}\rangle =0.5$, *T* = 300 *K*, *χμ*_*o*_ = 1.26⋅10^−6^ *N A*^−2^ and *M*_*s*_ = 10^6^ *A m*^−1^, extensions of 2% and 20% are required for particles with diameters of 200 and 50 nanometers, respectively. In any case, if there are limitations for the increase of the size of the magnet, the selection of a magnet with a larger magnetization can be an alternative option to satisfy Eq 21.

**Fig 5:**
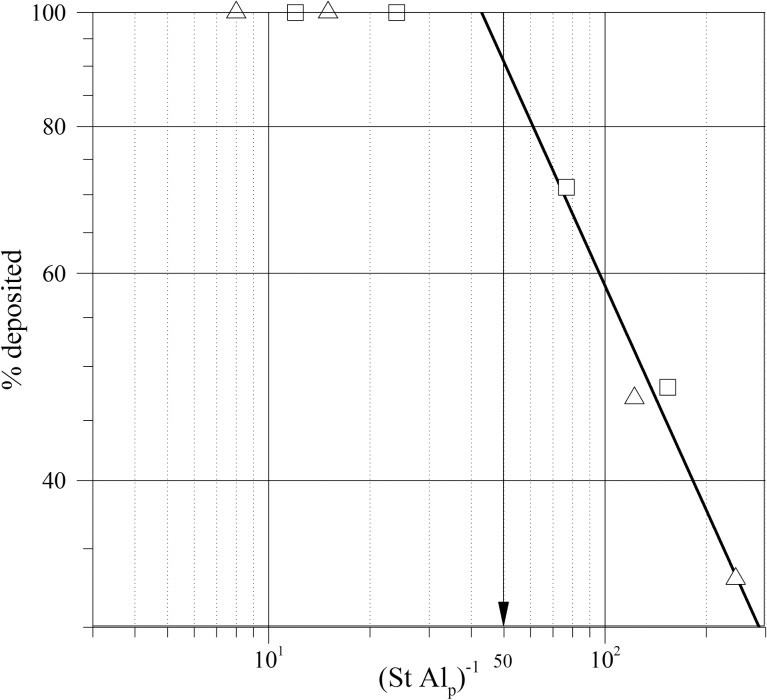
Fraction of particles deposited as a function of the parameter 1/(*St Al*_*p*_). Triangles correspond to the lateral position of the magnet and squares to the centered position.

Numerical simulations of the trajectories of the particles have been carried out for the lateral position of the magnet (cases L1 to L4) and for central position (cases C1 to C4). The physical parameters and the conditions considered for the simulations are summarized in Tables 1 and 2. Table 2 shows that in cases L1, L2, C1 and C2, *L*_*m*_/*h* is larger than 1/(*St Al*_*p*_) and, in agreement with Eq 17, all the particles initially released at the inlet of the microchannel are deposited on the walls. Fig 5 shows the fraction of deposited particles as a function of the parameter 1/(*St Al*_*p*_). It can be seen that for 1/(*St Al*_*p*_) < *L*_*m*_/*h* = 50 all the particles are deposited. For 1/(*St Al*_*p*_) > 50 a potential decrease of the fraction of deposited particles is observed. The potential fitting shown in Fig 5 predicts 90% of deposition at 1/(*St Al*_*p*_) = 50.

**Table 1:** Physical parameters.

*d*_*p*_	*ρ*_*p*_	*ρ*_*f*_	*μ*	*L*_*m*_	*h*	*W*	$\bar{V}$
[m]	[kg m^-3^]	[kg m^-3^]	[Pa s]	[m]	[m]	[m]	[m s^-1^]
10^−6^	1.6·10^3^	10^3^	10^−3^	5·10^−3^	10^−4^	5·10^−4^	0.1–0.01

**Table 2:** Non-dimensional parameters used in the simulations and predicted fraction of deposited particles.

Case	*Fr*	*M*	*Re*	*St*	*Al*_*p*_^(1)^	*L*_*m*_/*h*	1/*StAl*_*p*_	% deposited
L1	3.68	2.49 10^4^	1	8.89 10^−6^	9.21 10^3^	50	12	100
L2	3.68	1.24 10^4^	1	8.89 10^−6^	4.60 10^3^	50	24	100
L3	3.68 10^−2^	2.49 10^2^	10	8.89 10^−5^	9.21 10^1^	50	122	47
L4	3.68 10^−2^	1.24 10^2^	10	8.89 10^−5^	4.60 10^1^	50	244	33
C1	3.68	2.49 10^4^	1	8.89 10^−6^	1.47 10^4^	50	8	100
C2	3.68	1.24 10^4^	1	8.89 10^−6^	7.47 10^3^	50	15	100
C3	3.68 10^−2^	2.49 10^2^	10	8.89 10^−5^	1.47 10^2^	50	77	71
C4	3.68 10^−2^	1.24 10^2^	10	8.89 10^−5^	7.33 10^1^	50	154	48

^(1)^The volume averaged non-dimensional magnetic force, $\langle \left({\vec{B}}^{*}\cdot {\vec{\nabla }}^{*}\right)B_{z}^{*}\rangle$, is 0.37 for the lateral cases and 0.59 for the centered cases (*L*_*m*_ = 5 mm and *W* = 0.5 mm) according to Fig 3.

**Fig 6:**
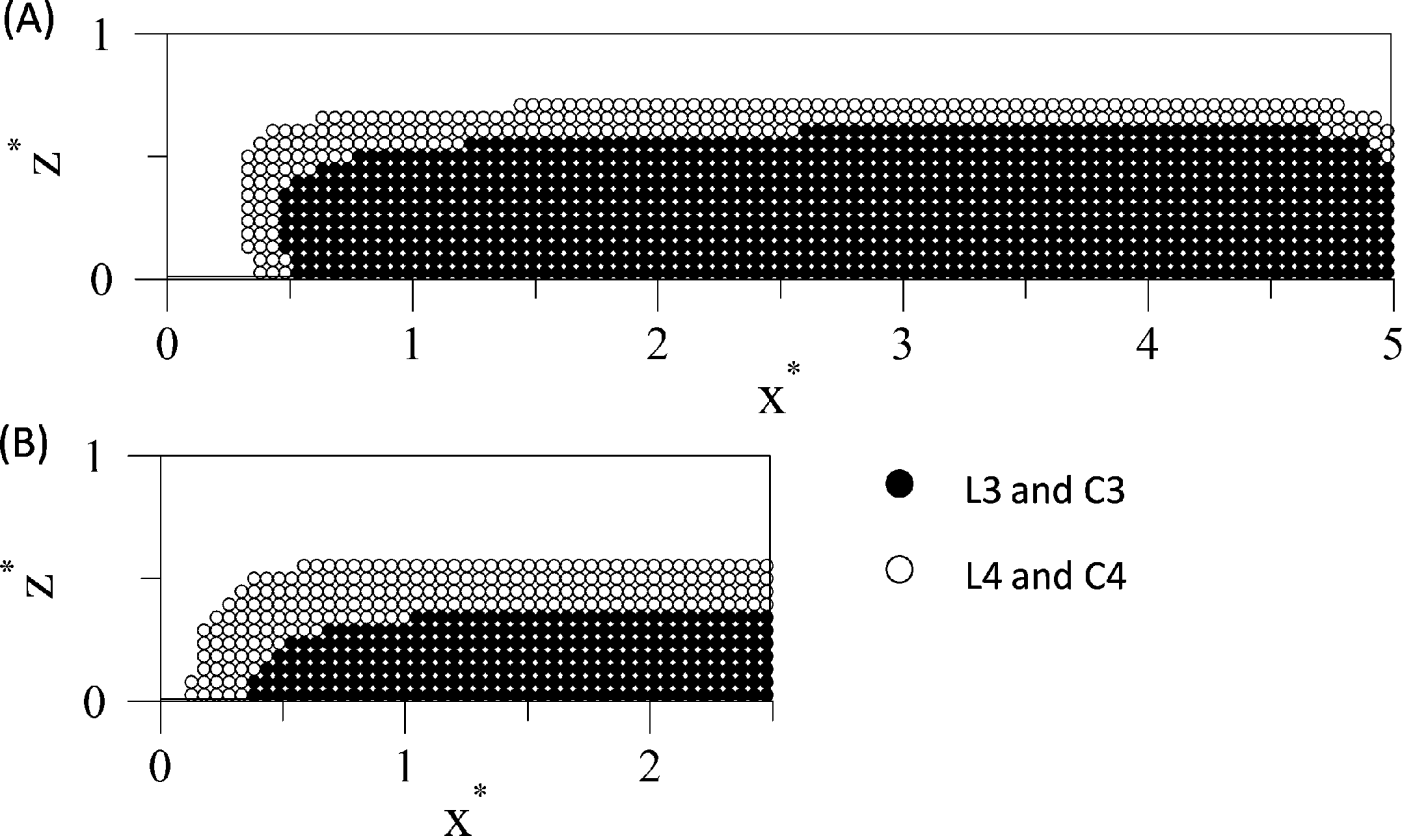
Initial positions of the particles that are not deposited on the walls of the microchannel. (A) Lateral position. (A) Central position (because of symmetry only half of the section is shown). Filled symbols correspond to cases L3 and C3 and open symbols to cases L4 and C4 (see Table 2).

In Cases L3, L4, C3 and C4 only a fraction of the particles is captured by the magnet as indicated in Table 2. Fig 6 marks the initial positions of the particles that are not deposited on the walls and consequently they leave the computational domain through the outlet of the microchannel. It can be seen for the lateral cases L3 and L4 (Fig 6A) that the magnet deposits the particles initially located close to the top and to the right lateral walls. In Cases C3 and C4 (Fig 6B), in which the magnet is centered with respect to the microchannel, the captured particles are initially located close to the top and to both lateral walls because of the relatively low velocity of the flow near these lateral walls.

**Fig 7:**
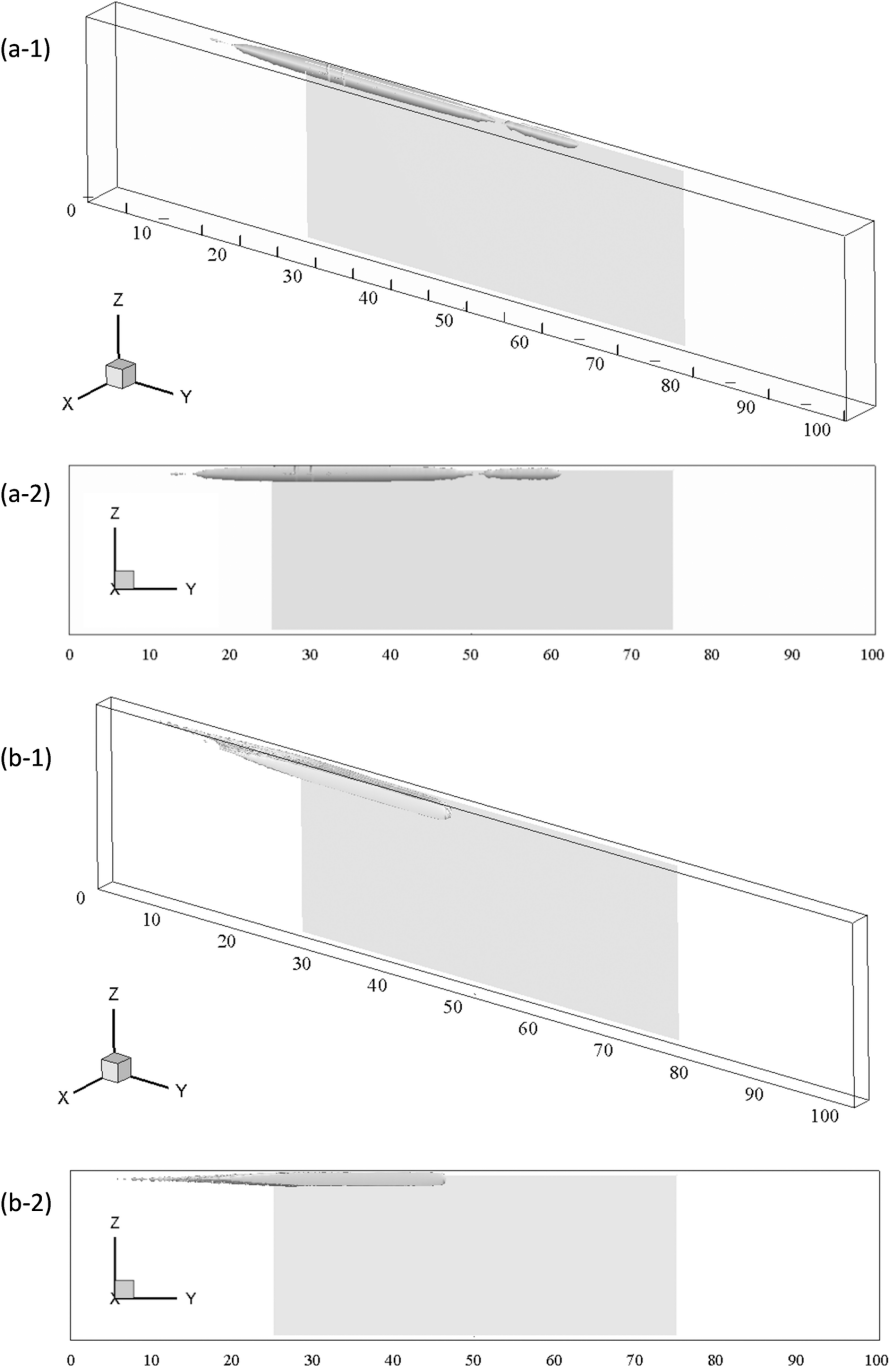
Isosurfaces of particle concentrations. (A) Case L1. (B) Case C1 (see Table 2). The vertical scale of the channel has been enlarged for better visualization and the projection of the magnet located between 25<x^*^<75 is shown in grey.

Fig 7 shows the regions with high rates of particle deposition for cases L1 (Fig 7A-1 and 7A-2) and C1 (Fig 7B-1 and 7B-2) for which all the particles are deposited on the wall. The time is larger than 525 non-dimensional units which is large enough to allow the major part of the particles to reach their final location of deposition (i.e. one throughflow corresponds to 100 non-dimensional time units). To compute the deposition rate the computational domain has been divided into 49x499x19 equal volumes for the lateral case and into 99x499x19 equal volumes for the centered case. Note that initially a particle is located in the center of the volumes situated at the inlet of the computational domain. At each time step the number of new particles in each volume is stored and summed up during the simulation. Thus, this quantity represents the rate of increase of the number of particles in each volume per time step, or equivalently, the rate at which the concentration of particles increases with respect to the concentration of particles at the inlet per time step. The isosurfaces plotted in 7Fig A and 7B correspond to a value of 16 and 6, respectively. In the case of the lateral position of the magnet (Fig 7A) the particles are deposited near the lateral wall closest to the magnet, while for the centered position (Fig 7B) the particles are deposited on the top wall. In both cases the simulations predict the location of the accumulations below leading edge of the magnet near the first half of the streamwise length of the magnet.

## Conclusions

In this study we analyzed numerical simulations of the trajectories of magnetic beads in a straight microchannel under the influence of a nearby permanent cubical magnet for different flow and magnetic conditions. Analytically derived local fluid velocities and local magnetic forces have been used to track the particles. A centered position and a lateral position of the magnet above the microchannel are considered. It has been found theoretically that the fraction of the particles deposited on the walls of the microchannel depends on the Stokes and Alfvén particle numbers and that the size of the magnet should be larger than the height of the microchannel divided by the Stokes and Alfvén particle numbers to capture all the particles uniformly distributed across the section of the microchannel. The results of the numerical simulations are in agreement with this criterion. The Lagrangian tracking of the particles has shown that accumulations of deposited particles occur on the top wall of the microchannel for the centered position of the magnet, while for the lateral position the accumulation is located on the lateral wall closest to the magnet. In both cases the simulations predict the location of the accumulations below leading edge of the magnet near the first half of the length of the magnet.

Greek letters

Subscripts

Superscripts

## Nomenclature

Al: Alfvén number, $Al=B^{2}/\mu_{o}\rho {\bar{V}}^{2}$
Al_p_: Particle Alfvén number (see Eq: 17: )
B: Magnetic flux density [T]
C_D_: Drag coefficient
d: Diameter [m]
D_Br_: Brownian diffusion coefficient [m^2^ s^-1^]
F: Force [N]
Fr: Densimetric Froude number $Fr=\bar{V}/{\left(gh\;(1-\rho_{f}/\rho_{p})\right)}^{1/2}$
g: Gravity acceleration [m s^-2^]
h: Height of the microchannel [m]
k_B_: Boltzmann constant (*k*_*B*_ = 1.381·10^−23^ m^2^ kg s^-2^ K^-1^)
L: Length [m]
m: Mass [kg]
M: Non-dimensional group (see Eq: 11: )
M_s_: Magnetization of the magnet [A m^-1^]
r: Position vector [m]
Re: Reynolds number, $Re=\frac{\bar{V}h}{\nu }$
Re_p_: Particle Reynolds number, $Re_{p}=\left|{\vec{V}}_{p}-\vec{V}\right|d_{p}/\nu$
St: Stokes number, $St=\rho_{p}d_{p}^{2}\bar{V}/\left(18\;\mu h\right)$
t: Time [s]
T: Temperature [K]
u,v,w: Velocity vector components [m s-1]
V: Velocity [m s-1]
W: Width of the channel [m]
x,y,z: Cartesian coordinates [m]
μ: Dynamic viscosity [Pa s]
μ_o_: Permeability of vacuum (*μ*_*o*_ = 4π·10^−7^ N A^-2^)
ν: Kinematic viscosity [m^2^ s^-1^]
ρ: Density [kg m^-3^]
σ: Standard deviation of the Brownian displacements [m]
χ: Magnetic susceptibility
p: Particle
f: Fluid
m: Magnet, magnetic
*: Dimensionless quantity
